# Supplementary material for: A Global Screen for Assembly State Changes of the Mitotic Proteome by SEC-SWATH-MS
Source: Cell Syst. 2020 Feb 26;10(2):133–155.e6. doi: 10.1016/j.cels.2020.01.001 (PMC7042714; doi:10.1016/j.cels.2020.01.001)

Q9GZP4 | PITH1_HUMAN | PITHD1 C1orf128 AD039 HT014 PP603
Monomer MW [kDa]: 24.178 Monomer expected elution fraction: 53

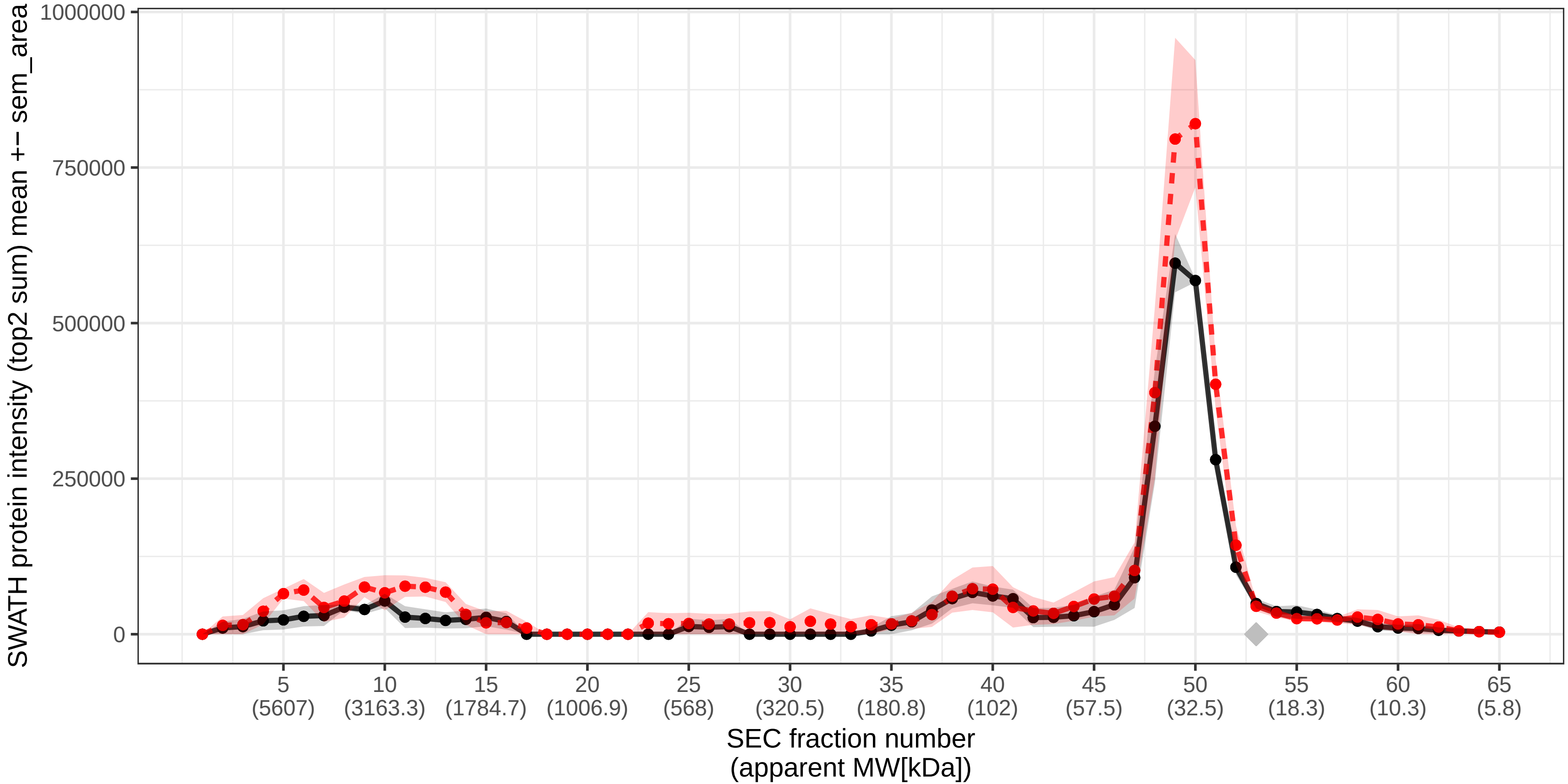

Supplement: Data S1. SEC-SWATH-MS Protein Chromatograms, Related to Figure 1 [file mmc6.zip › SECchrom_Q9GZP4_PITH1_HUMAN_PITHD1_C1orf128_AD039_HT014_PP6.pdf]
